# Nitrogen balance and efficiency as indicators for monitoring the proper use of fertilizers in agricultural and livestock systems

**DOI:** 10.1038/s41598-022-15615-7

**Published:** 2022-07-14

**Authors:** Joyce Graziella Oliveira, Mário Luiz Santana Júnior, Nayane Jaqueline Costa Maia, José Carlos Batista Dubeux Junior, Augusto Hauber Gameiro, Taise Robinson Kunrath, Gabriela Geraldi Mendonça, Flávia Fernanda Simili

**Affiliations:** 1 Instituto de Zootecnia/APTA/SAA, Ribeirão Preto, SP 14030-670 Brazil; 2 Instituto de Zootecnia/APTA/SAA, Nova Odessa, SP 13460-000 Brazil; 3 Faculdade de Ciências Agrarias e Veterinárias (FCAV), Universidade do Estado de São Paulo (UNESP), Jaboticabal, SP 14884-300 Brazil; 4 University of Florida-IFAS, North Florida Research and Education Center, 3925, Highway 71, Marianna, FL 32446 USA; 5 Faculdade de Medicina Veterinária e Zootecnia (FMVZ), Universidade de São Paulo (USP), Pirassununga, SP 13635-900 Brazil

**Keywords:** Environmental impact, Forestry

## Abstract

The rational use of nutrients is a key factor for the sustainability of agricultural systems. This study aimed to analyze the nitrogen balance and use efficiency, and the valorization of organic residues within integrated systems, in comparison to conventional agricultural and livestock systems. The experiment was assembled in a randomized blocks design with three replicates. Six production systems were compared, grain maize production (CROP) and pasture for beef cattle production (LS), and four ICLS (Integrated Crop-Livestock System) for grain maize and pastures for beef cattle, in 2 years. In order to estimate the nutrients balance, inputs, and outputs at farm levels were considered, and with the results obtained for nutrient balance, the use efficiency was calculated. The CROP presented higher nutrient use efficiency (1.43 kg/ha^−1^), but at the same time, it resulted in negative contributions for the nutrient balance (−97 kg/ha^−1^) because of lower amounts of nitrogen in the organic residues (188 kg/ha^−1^) and lower valuation. The LS and ICLS provided a higher amount of nitrogen (983 kg/ha^−1^; mean ± 921 kg/ha^−1^) and valuation of organic residues. The presence of components such as pastures and the animal contribute to a positive production system, while reducing the needs for chemical fertilizers.

## Introduction

The anthropogenic production of nitrogen fertilizers requires elevated energy inputs^1^ and, in recent decades, there has been a worldwide increase in the production of these fertilizers by almost fivefold^2^. According to FAO^3^, more than 109 million tons of nitrogen fertilizers were used for agricultural and livestock production in 2017. The importance of using nitrogen fertilizers in agriculture is due to its fundamental roles in plants growth^4,5^. In addition of being the most limiting nutrient in crop systems, due to its high exportation in crops^4^. However, the non-rational use of nitrogen in agricultural production systems can compromise crops’ yield and cause environmental and soil damages^6^.

Reaching an equilibrium of nutrients in agriculture and livestock production systems is a challenge^4^. Therefore, adopting systems that integrate both activities could represent a more sustainable alternative to conventional systems, considering that a synergistic interaction between systems can be achieved, thus optimizing the use of fertilizers when producing goods. The integrated systems are commonly used in some countries^7–9^. However, countries such as Brazil has large areas of crops and pastures for cattle, and these systems have moderate representativeness in relation to total production^10–12^.

Recent research has demonstrated the benefits of integrated systems in comparison to conventional ones, and these benefits include mitigate environmental trade-offs^12^, nutrient cycling^10,13,14^, the acquisition of more than one product per unit of area^15^, improvements of soil properties^10^, reductions in the use of fertilizers^13^ and sharing of inputs between crops^16^. Over the years, it was possible to observe a synergism among the soil–plant–animal components of production systems, due to a greater nutrient cycling caused by the presence of animals^17^, thus contributing for the reduction on the use of synthetic fertilizers. The implementation of Integrated Crop-Livestock Systems (ICLS) can be carried out via intercropping, between grain-producing crops and pastures for cattle production, which can be presented in assembled arrangements, as a function of species, spacing and planting techniques^18^. Thus, despite the benefits, the hesitation to implement ICLS has been based on the complexity of the system, need for machinery for both activities, qualified labor and management^11^. Sowing methods and the consortium between species can affect soil quality^18^ and possible the nutrient balance (NB) and nutrient use efficiency (NUE), which are well-known approaches used for nutrients management in agricultural and livestock systems^7,19^.

For instance, it is possible to estimate the deficit or surplus of nutrients using the NB^20^, which, in a simplified way, is the difference between nutrients inputs and outputs in the system^20,21^. The NB is an agri-environmental indicator that helps monitoring the nutrient flow, contributing in a positive way for the rational use of mineral and organic fertilizers^7,22^. Based on the NB data, the NUE is considered a dimensionless indicator, being calculated as the ratio between outputs and inputs of nutrients in a production system^19^.

The main components that determine both NB and NUE are the nutrients inputs and outputs in the system. However, there is little information regarding this study area, and in addition, the methodologies used to calculate the NB and NUE in systems were not standardized. The equilibrium between nitrogen inputs and outputs, as well as its transformations over time, are essential traits that provide adequate amounts of nutrients in production systems^23^.

According to Gameiro et al.^24^ and Gerber et al.^19^, the management of natural resources and nutrients flow are increasingly focused on the concept of food production efficiency. In relation to the agricultural system, this synergism and management is highly important, as at the same time, increase productivities and environmental sustainability.

In view of this scenario, the aim of this study was to use and evaluate NB and NUE as indicators for monitoring the use of nitrogen in integrated systems, in comparison to conventional systems of agricultural and livestock production. The indicators were also used to compare different sowing methods for the implementation of ICLS, aiming to evaluate if managements practices interfere in the NB and NUE. In addition, calculations were carried out to estimate the valuation of the organic residues generated in these systems. The hypothesis of the present study is that ICLS contributed positively for the balance and use efficiency of nitrogen, which might lead to the reduction on the use of synthetic fertilizers and, use of NB and NUE is an efficient tool to improve agricultural systems.

## Results and discussion

### Estimate of the nutrient balance (NB) and nutrient use efficiency (NUE)

The NB indicator showed significantly negative results for CROP in relation to other treatments (p < 0.0001), possible due to a higher export of N (output) (Table 1), and as a function of the high demands that maize crops have for grain production.

**Table 1:** Estimate of the nutrient balance (NB) and nutrient use efficiency (NUE), kg ha^−1^, in two experimental years.

	Treatments						*p*
	CROP	LS	ICLS-1	ICLS-2	ICLS-3	ICLS-4	
**Input (kg ha**^**−1**^**)**							
Fertilizer	224	112	192	192	192	192	
**Output (kg ha**^**−1**^**)**							
Products	321a	18c	172b	190b	180b	188b	< 0.0001
NB	−97c	94a	20b	2b	12b	4b	< 0.0001
NUE	1.43a	0.16c	0.89b	0.99b	0.94b	0.98b	< 0.0001
SN (soil)	11570b	11050b	14570ª	13750ª	11190b	14370a	< 0.0001

Means followed by distinct letters are statistically different, according to the Tukey’s test at a 5% probability level.

*CROP* production of maize grain, *LS* production of beef cattle in pasture, *ICLS-1* maize and Marandu grass sowed simultaneously without herbicide, *ICLS-2* maize and Marandu grass sowed simultaneously with herbicide, *ICLS-3* delayed sowing of maize and Marandu grass, *ICLS-4* maize and Marandu grass sowed simultaneously in maize lines and interlines with herbicide, *SN* soil nitrogen stocks.

The nutrient input for grain production presented distinct translocation rates in the tissue, which was considered high in relation to the export of N to the grain, on average 73%^25,26^. On one hand, this result showed that the application rate used with the mineral fertilizer did not provide sufficient amounts of the nutrient to achieve a satisfactory production of grains. According to Galindo et al.^6^ the availability of nutrients in soil varies based on how residue is managed and also the amounts of N that are applied.

On the other hand, the NB was positive in treatments that had the animal component, with higher values being observed for LS in comparison to integrated systems, which were statistically similar among each other (p < 0.0001). The NB in LS was significantly higher in comparison to all other treatments (p < 0.0001), which might indicate that in systems where beef cattle is reared in exclusive pasture, the need for N fertilization is lower in comparison to others, possibly due to a low demand of N for animal production, in relation to an agricultural system^21,27^. These results are similar to those reported by Ryschawy et al.^28^, in which the authors verified a negative balance for crop (−11.9 ± 34.2 kg ha^−1^ of N), and a positive balance for beef farm (37.9 ± 23.3 kg ha^−1^ of N), throughout one year of evaluation, without considering the stock of N in the soil.

Integrated systems presented more balanced results in comparison to conventional systems, because while they were efficient and presented a NUE varying from 0.89 to 0.99, they managed to maintain the NB positive, with a little surplus of N and without having to appeal to the soil’s emergency reserve (Table 1). Alvarez et al.^1^ Tadesse et al.^8^ and Zingore et al.^9^ and also verified positive NBs in integrated systems, with respective values of 94, 38, and 21 kg N ha^−1^ year^−1^.

There is a concern about the excess of N in the soil could be harmful to production systems, thus the estimates for the calculation of NB are important to understand nutrient flow and mineral fertilizer supply. Some authors reported that depending on the type of soil and climate, N losses can be higher, or this surplus can be reused in cycles^29,30^.

The NUE is directly related to the sustainability of the production system^31^. Thus, an efficient use of the nutrient is essential for the synchrony between the nutrient released by fertilizers and the crop demand, otherwise losses might occur^32^. The indicator NUE showed higher efficiency of N use for CROP in comparison to other treatments, but in the LS it was significantly lower in comparison to the integrated systems, which did not differ among each other (p < 0.0001, Table 1).

Although the CROP system was more efficient in using the N derived from the mineral fertilizer, it was necessary to use the soil emergency reserve of this nutrient. The crop’s demand for this nutrient was possibly met via N stock as an emergency reserve, in order to guarantee a high production of grains (Table 2), which in the long-term could result in a severe extraction and depletion of the soil N, in case there is no replacement of this macronutrient in the system^27,33^. According to Van Raij et al.^27^, maize is one of the most demanding crops in relation to soil fertility, making the supply of nutrients essential to achieve satisfactory results. A study on maize roots demonstrated that when the concentrations of nitrate were high in the soil, as a function of an excessive fertilization rate, roots did not develop well^34^. However, when the nutrient was supplied in sufficient amounts, an ideal lateral development of roots was verified, which can thus be a promising way of increasing the NUE when N is added to the soil^34^.

**Table 2:** Data the maize grain, animal tissue (Tissue), litter deposited (LD); animal manure and stocking rate (SR) in the agricultural systems during two years of experiment.

	Treatments (kg ha^−1^)						*p*
	CROP	LS	ICLS-1	ICLS-2	ICLS-3	ICLS-4	
Grain	20.947ª	–	10.980b	12.068b	11.412b	11.867b	< 0.0001
Tissue	–	41.000	65.000	81.000	86.000	97.000	0.1902
Straw	14.245ª	–	9.784ab	7.574b	6.437b	7.763b	0.0044
LD	–	38.877	34.695	33.792	32.209	34.636	0.1926
Urine	–	37.283a	29.653b	28.867b	29.051b	30.438b	< 0.0001
Feces	–	55.630a	44.245b	43.073b	43.347b	45.417b	< 0.0001
SR		3.780a	3.280b	3.210b	3.220b	3.350b	< 0.0001

Means followed by distinct letters are statistically different, according to the Tukey’s test at a 5% probability level.

*CROP* production of maize grain, *LS* production of beef cattle in pasture, *ICLS-1* maize and Marandu grass sowed simultaneously without herbicide, *ICLS-2* maize and Marandu grass sowed simultaneously with herbicide, *ICLS-3* delayed sowing of maize and Marandu grass, *ICLS-4* maize and Marandu grass sowed simultaneously in maize lines and interlines with herbicide.

Therefore, we emphasize that soil fertility conditions could result in greater use by shoots and roots of maize^35^. Generally, an increased rate of N fertilization increases grain productivity. However, this does not mean that the more N applied, the greater the grain yield that can be achieved^33^. This fact corroborates the results presented by Gerber et al.^19^, who reported that the evaluations of NUE in production systems are more challenging when only the animal component of the system is evaluated.

### Quantity of nitrogen and valuation of organic residues

The amount of N in the straw and animal excreta (urine and feces) were statistically different among treatments (Table 3). Higher concentrations of N in the straw were verified in the CROP treatment, because of the two harvests of maize during the experimental years (Table 2), while higher N contents in the animal excreta were found for LS (p < 0.0001, Table 1), due to a higher stocking rate (p < 0.0001, Table 2). However, the amount of N in the LD did not differ statistically among treatments (p = 0.3227, Table 3), considering that the amount of LD in treatments with pasture and cattle was similar (Table 2).

**Table 3:** Amount of nitrogen (kg ha^−1^) and valuation of organic residues.

	Treatments						
	CROP	LS	ICLS-1	ICLS-2	ICLS-3	ICLS-4	*p*
Straw	188.03a	–	129.15ab	99.98b	84.97b	102.47b	0.0035
LD	–	436.49	383.51	377.31	393.78	384.61	0.3227
Urine	–	428.75a	341.01b	331.97b	334.09b	350.04b	< 0.0001
Feces	–	117.74a	93.64b	91.16b	91.74b	96.12b	< 0.0001
Org Res	188.03b	982.98a	947.31a	900.43a	904.58a	933.24a	< 0.0001
L. Volat	52.65b	275.24a	265.25a	252.12a	253.28a	261.31a	< 0.0001
Urea	300.86b	1572.78a	1515.69a	1440.69a	1447.32a	1493.18a	< 0.0001
Value ($)	84.15b	439.89a	423.93a	402.95a	404.80a	417.63a	< 0.0001

Means followed by distinct letters are statistically different, according to the Tukey’s test at a 5% probability level.

Average tonne of urea (2010–2020); kg urea = $0.28 (Source: World Bank Price Data).

Value: estimated value of organic residues.

*CROP* production of maize grain, *LS* production of beef cattle in pasture, *ICLS-1* maize and Marandu grass sowed simultaneously without herbicide, *ICLS-2* maize and Marandu grass sowed simultaneously with herbicide, *ICLS-3* delayed sowing of maize and Marandu grass, *ICLS-4* maize and Marandu grass sowed simultaneously in maize lines and interlines with herbicide, *LD* litter deposited, *Org Res* organic residue, amount of nitrogen in straw, litter deposited, urine and feces, *L. volat* losses of nitrogen by volatilization, *urea* equivalent to the urea fertilizer, with 45% of N.

The reuse of nutrients via animal production is one of the main advantages of systems containing cattle in pastures. According to Dubeux Jr. and Sollenberger^23^, ruminants return between 80 and 90% of the nutrients consumed in the system via their excreta. The results presented in this study showed higher values of N in the organic residues of treatments that had cattle inserted, on average five times more than the CROP system (Table 3), even when considering the losses of N by volatilization (28%).

In a study conducted in a tropical region, Rodrigues et al.^36^ verified a return of 73 kg ha^−1^ of N via excreta in a livestock system, while in this study we observed mean values of 546 kg ha^−1^ of N for the soil in the LS treatment (Table 3). During two years of experiment, the average amount of N excreted via urine and feces in the ICLS treatments were 339 and 93 kg ha^−1^, while in the LS the excretion was on average 429 and 118 kg ha^−1^, respectively. Thus, the return of N to the pastures averaged 432 kg ha^−1^ in ICLSs and 546 kg ha^−1^ in LS. In both treatments, the return of N to the soil was 79% via urine and 21% via feces. The highest amounts of N, referring to the total accumulated, were higher in LS and ICLSs (p < 0.0001, Table 3) evidencing a greater potential of N cycling in the treatments containing cattle raised in pastures.

The potential to reuse the nutrients excreted by cattle in livestock systems is high^7^. Svanbäck et al.^37^ reported that the use of nutrients from animal excreta is more efficient in order to reduce the need for mineral fertilizers, which consequently contributes for the economic feasibility of agricultural and livestock systems.

The highest SR and number of grazing cycles in the LS treatment in relation to ICLS (p < 0.0001, Table 2) explain the greater amounts of N supplied by urine and feces in this system (p < 0.0001, Table 3). An increased SR can provide greater nutrient flow caused by the excreta^23^, which in turn rises the potential for nutrients cycling in this system. However, it is important to note that an increased stocking rate might cause negative damages to the soil, leading to greater nutrient losses by erosion or leaching^38,39^.

We observed that both the LS and ICLS treatments provided a higher valuation of organic residues in comparison to the CROP system (p < 0.0001, Table 3). In this sense, the presence of pastures and the animal component in the system can contribute in a positive way to the inputs of N in production systems, while reducing the needs for chemical fertilizers and the occurrence of environmental issues. In addition, according to Hong et al.^40^, an efficient use of animal excreta can largely meet the nutrient requirements of agricultural and forage crops.

In integrated systems, the production of grains starts to benefit by the presence of animals, due to the synergism between cycling components^17,41^, as the quality of the residue and the inclusion of animals in the system will dictate the proportion and species of the associated microbiota that will act in the nutrient cycling in subsequent crops^17^.

The sowing methods adopted when implementing the ICLS treatments did not affect the NB and NUE (Table 1), as well as the amount of N in the organic residues and the residue valuation (Table 3). However, our results indicate that ICLSs contribute positively for a greater valuation of organic residues in comparison to the CROP system, demonstrating that the residues from the intercropping in these systems are of paramount importance for the balance of N, as well as for reductions in the use of fertilizers in agricultural systems.

## Materials and methods

### Site description

**Figure 1:**
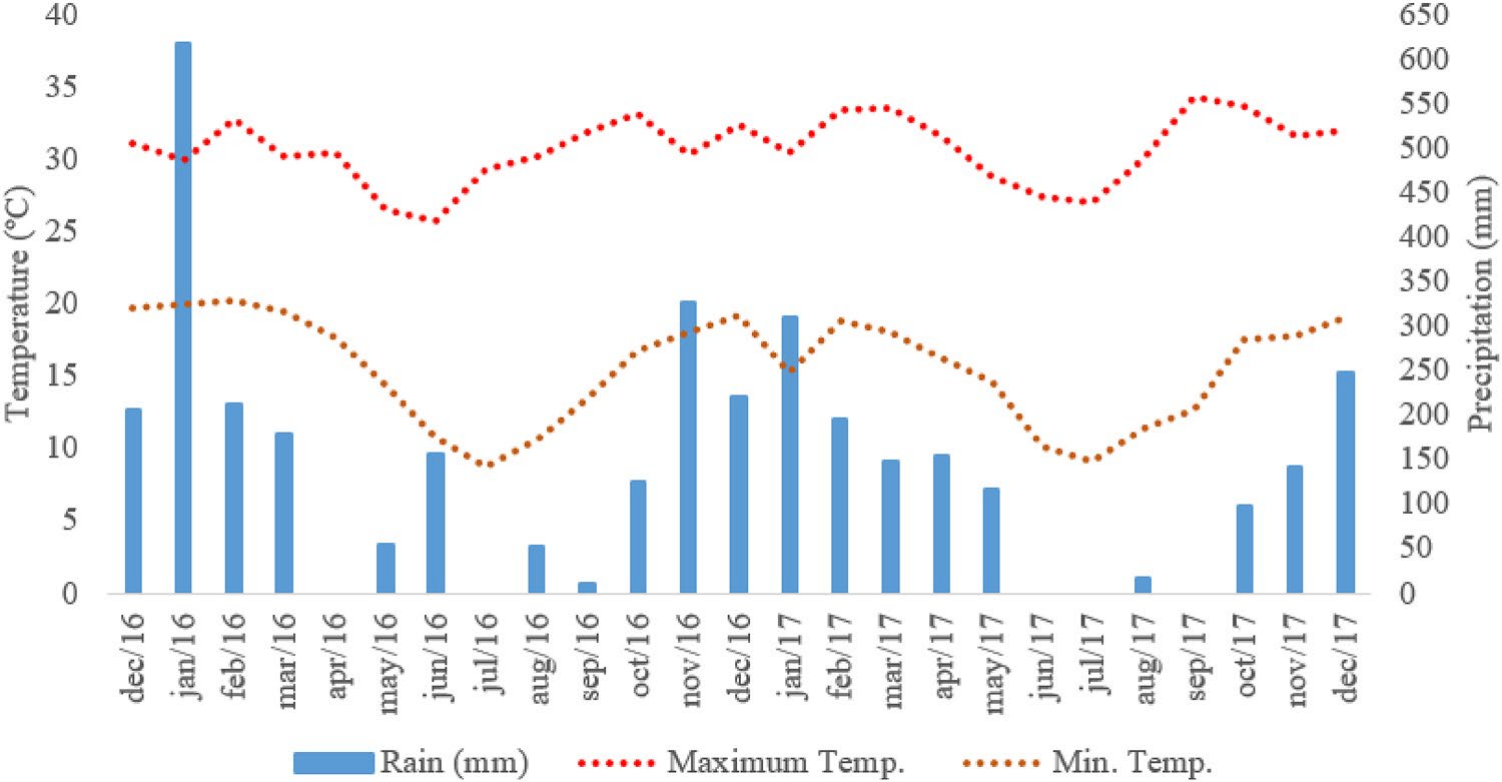
Meteorological data during the study period, obtained from the meteorological station located at Centro de Pesquisa de Bovinos de Corte, Instituto de Zootecnia/Agência Paulista de Tecnologia dos Agronegócios (APTA)/Secretaria de Agricultura e Abastecimento de São Paulo (SAA), Sertãozinho, São Paulo, Brazil.

The experiment was conducted at the Beef Cattle Research Center of the Institute of Animal Science/APTA/SAA, Sertãozinho, São Paulo, Brazil (21°08′16″ S e 47°59′25″ W, average altitude 548 m), during two consecutive years. The climate in this region is Aw according to the Köppen’s classification, characterized as humid tropical, with a rainy season during summer and drought during winter. The meteorological data is reported in Fig. 1. The soil in the experimental area is classified as an Oxisol^42^. Before the experiment, soil samples were collected for chemical characterization (Table 4), which was performed following the methodology described in Van Raij et al.^43^. Samples were collected in 18 experimental paddocks, at the depths of 0- to 10- and 10- to 20-cm layers, from 10 distinct sampling points in each paddock, in order to create one composite sample per unit, totaling 36 samples analyzed.

**Table 4:** Chemical attributes of the soil in the experimental area, before installing the experiment (November 2015).

Depth	P resin	SOM	pH	K^+^	Ca^2+^	Mg^2+^	CEC	BS
Cm	mg dm^-3^	g dm^-3^	CaCl_2_	mmolc dm^-3^				%
0–10	14	31	5.2	3.1	25	15	79	54
10–20	9	30	4.9	1.8	10	12	78	42

*P resin*  phosphorus determined by the resin method, *SOM* soil organic matter, *pH*  active acidity, *K*  exchangeable potassium, *Ca*  exchangeable calcium, *Mg*  exchangeable magnesium, *CEC*  cation exchange capacity, *BS*  bases sum.

The nitrogen total (Nt) content was determined by the micro-Kjeldahl method^44^, and the soil nitrogen stocks (SN) were calculated using the following equation below, according to Veldkamp et al.^45^.

$${\text{SN }}\left[ {{\text{Mg ha}}^{ - 1} {\text{ at a given depth}}} \right]\, = \,({\text{concentration }} \times {\text{ BD}}\, \times \,{1}/{1}0),$$

where concentration refers to the Nt concentration at a given depth (g kg^−1^), BD is the bulk density at a certain depth (average 1.24 kg dm^−3^), and 1 is the layer thickness (cm).

### Description of treatments and managements

**Figure 2:**
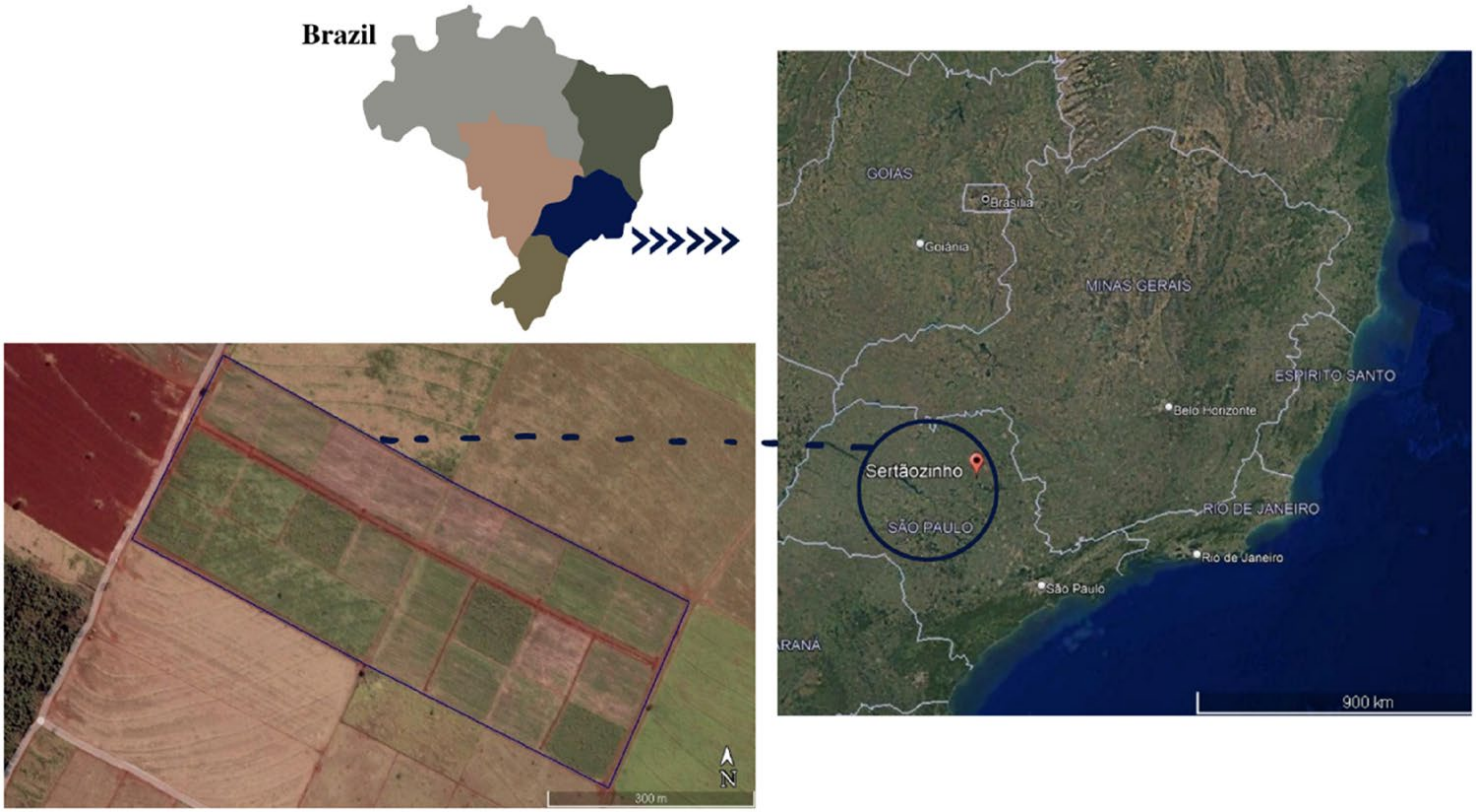
Localization and representation of the area of the experiment carried out in the study. Google Earth version Pro was used to construct the map (http://www.google.com/earth/index.html).

The experiment was carried out in a 16-ha area, divided into 18 paddocks of 0.89 ha each (Fig. 2), organized in a randomized blocks design with three replicates and six treatments, namely conventional crop system with grain maize production (CROP), conventional livestock system with beef cattle production in pasture using Marandu grass (LS), and four ICLS for the production of intercropped maize grain with beef cattle pasture. All production systems were sowed in December 2015, under a no-tillage system. The fertilization recommendations in the systems were based on the recommendation presented in the Boletim 100^46^.

In the CROP system, the maize Pioneer P2830H was cultivated, sowed in a spacing of 75 cm and sowing density of 70 thousand plants. Applications of 32 kg ha^−1^ of nitrogen (urea), 112 kg ha^−1^ of P_2_O_5_ (single superphosphate) and 64 kg ha^−1^ of KCl (potassium chloride) were performed. Complementarily, a topdressing fertilization was made using 80 kg ha^−1^ of nitrogen (urea) and 80 kg ha^−1^ of KCl. Sowing was carried out for two consecutive years (December 2015 and 2016), providing two harvests of maize grains (May 2016 and 2017), and between one harvest and the other, the soil remained in fallow without any cover crop. The total amount of fertilizer applied in two years was 224 kg ha^−1^ of nitrogen (urea), 224 kg ha^−1^ of P_2_O_5_ (single superphosphate) and 288 kg ha^−1^ of KCl (potassium chloride).

For the LS treatment, Urochloa brizantha (Hoechst. ex A. Rich) R.D. Webster cv. Marandu (syn. Brachiaria brizantha cv. Marandu) was sowed in a spacing of 37.5 cm, with a density of 5 kg ha^−1^ of seeds (76% of crop value) for the pasture assemblage. Marandu grass seeds were mixed with the planting fertilizer, applying 32 kg ha^−1^ of nitrogen (urea), 112 kg ha^−1^ of P_2_O_5_ (as single superphosphate) and 64 kg ha^−1^ of KCl. Applications of 40 kg ha^−1^ of nitrogen, 10 kg ha^−1^ of P_2_O_5_ and 40 kg ha^−1^ of KCl were also performed as topdressing fertilization in October 2016 and March 2017. 90 days after sowing, the pasture was ready to be grazed (March 2016). Three grazing periods were carried out in continuous stocking systems, with the first period between March and April 2016, the second period between August and October 2016 and the third between November 2016 and December 2017. The total amount for 2 years was 112 kg ha^−1^ of nitrogen (urea), 132 kg ha^−1^ of P_2_O_5_ (single superphosphate) and 144 kg ha^−1^ of KCl (potassium chloride).

The same cultivar, spacing, sowing density and fertilization rates described in the CROP treatment were used in all ICLS, as well as the same density of Marandu grass seeds and topdressing fertilization adopted in the pasture of the LS treatment. The total amount for two years was 192 kg ha^−1^ of nitrogen (urea), 132 kg ha^−1^ of P_2_O_5_ (single superphosphate) and 224 kg ha^−1^ of KCl (potassium chloride). In ICLS-1, Marandu grass was sowed in lines simultaneously with maize, while in ICLS-2, the sowing was also simultaneous, but the application of an under-dose of 200 mL of the herbicide Nicosulfuron was used, 20 days after seedlings emergence. In the ICLS-3, Marandu grass seeds were sown the time of topdressing fertilization of maize, thus the grass seeds were mixed with the fertilizer, and sowing was carried out in the interlines of maize, using a minimum cultivator. In ICLS-4, the sowing of Marandu grass was performed simultaneously with maize, but the grass seeds were sowed in both rows and inter-rows of maize, resulting in a spacing of 37.5 cm. In this treatment, the application of 200 mL of the herbicide Nicosulfuron was adopted, 20 days after seedlings emergence.

In all ICLS treatments, maize harvest was carried out in May 2016. Ninety days after harvesting the plants, the pastures were ready to be grazed. Therefore, two grazing periods were made in continuous stocking, being the first period between August and October 2016 and the second period between November 2016 and December 2017. The method for animal stocking in treatments LS and ICLS was continuous with a stocking rate (put and take) being defined according to Mott^47^. Caracu beef cattle with 14 months of age were used at the beginning of the experiment, with an average body weight of 335 ± 30 kg.

### Estimations of the nutrient balance (NB) and nutrient use efficiency (NUE)

In this study, the inputs and outputs of N were assessed at the farm level^48,49^. The NB was calculated by the equation below^19,45,50^.

$${\text{NB}}_{{\text{N}}} = {\text{ Input}}_{{\text{N}}} {-}{\text{ Output}}_{{\text{N}}}$$

As for the NUE, this parameter was evaluated as defined by the EU Nitrogen Expert Panel^51^, being calculated as the ratio between outputs and inputs of nitrogen.

$${\text{NUE}}_{{\text{N}}} = \, \left[ {{\text{Output}}_{{\text{N}}} /{\text{ Input}}_{{\text{N}}} } \right]$$

where NB is the nutrient balance, N is nitrogen, Input is the N concentration in the mineral fertilizer (urea), Output is the nitrogen concentration in export (maize grain and animal tissue), and NUE is the use efficiency of the nutrient.

The amount of N exported in maize grains, the grain production results (Table 2) were multiplied by the mean value of N, consulted in Crampton and Harris^52^.

In order to estimate the amounts of nutrient exported by the animals in their tissues, the values of live weight gain were considered [kg ha^-1^ of live weight (PV)] (Table 2), as well as the nitrogen values of the tissue, according to the methodology proposed by Rasmussen et al.^21^. Those authors reported that for animals weighting less than 452 kg/PV, it represents 2.7%, while heavier animals have a 2.4% nitrogen content representation of their body weight.

**Figure 3:**
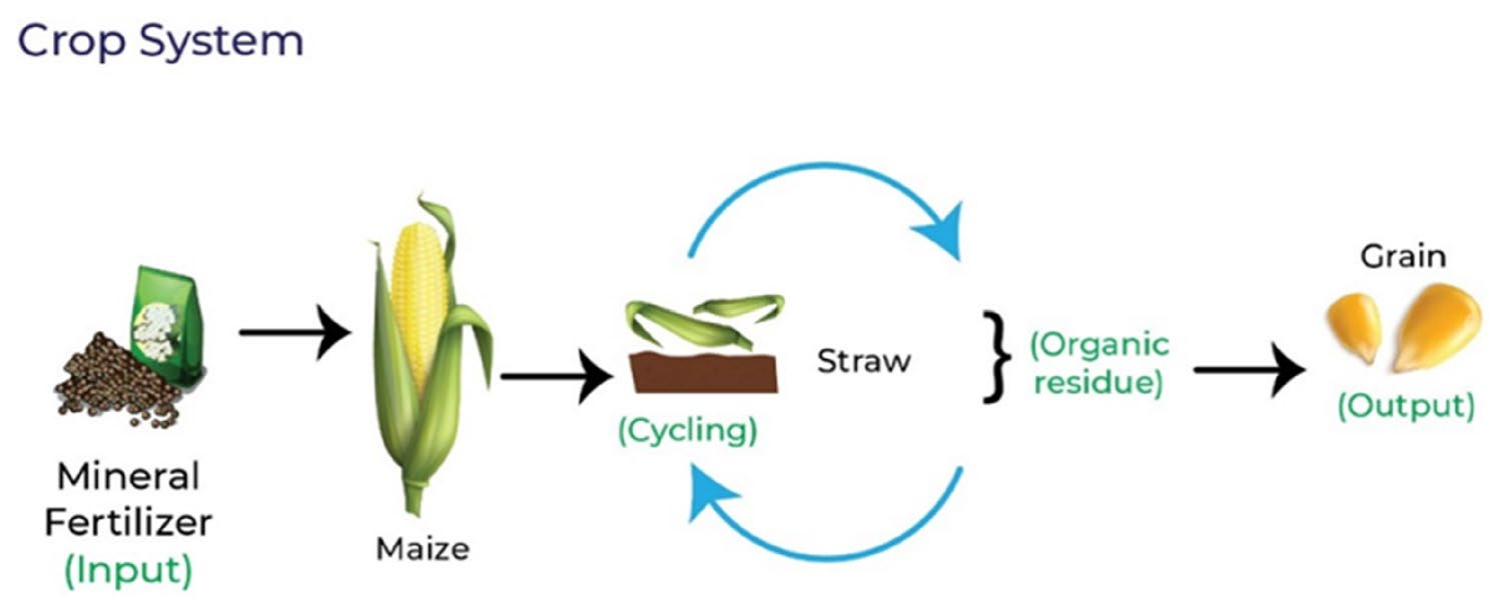
Representation of inputs and outputs of nitrogen and organic residues generated in the crop system.

**Figure 4:**
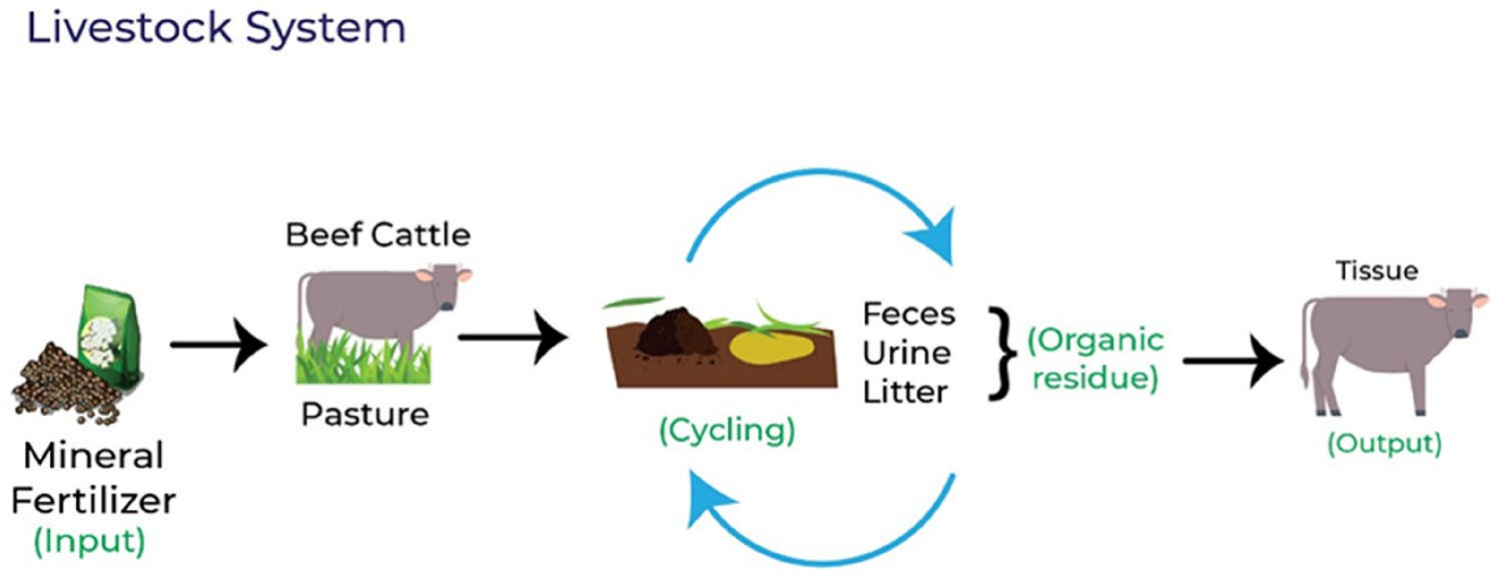
Representation of inputs and outputs of nitrogen and organic residues generated in the livestock system.

**Figure 5:**
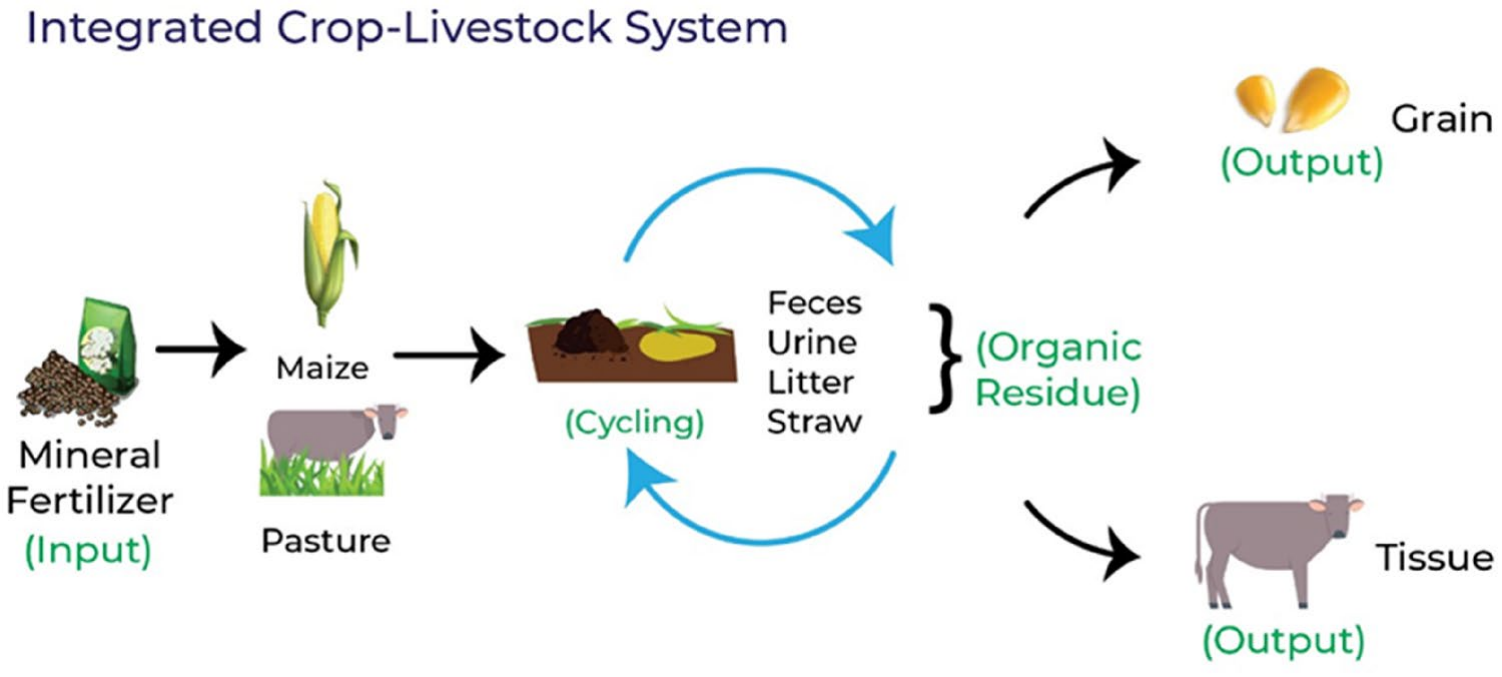
Representation of inputs and outputs of nitrogen and organic residues generated in the integrated systems.

The inputs and outputs of N in each production system are represented in Figs. 3, 4 and 5. Biological N fixation, atmospheric deposition, denitrification, leaching, rainfall, and volatilization and absorption of ammonia were not considered in the calculation of NB.

Data for animal tissue, animal excreta, and N concentration in grains were obtained from key manuscripts from the scientific literature in order to estimate the N balance.

### Calculation of nitrogen quantity and valuation of organic residues

The amount of N in the organic residues was determined as a function of the system (Figs. 3, 4, 5). The residue considered in the CROP was the straw derived from maize, while for LS it was the litter deposited (LD) in the grass Marandu, and animal manure (feces and urine). The ICLS were considered as the straw, LD, and animal manure.

The N concentration in straw and LD was determined following the methods of AOAC (1990). Straw was sampled immediately after maize grain harvest, using a 1-m^2^ frame in the field. The material was collected in two spots of the plot that were chosen randomly. All straw deposited on the soil was sampled, weighted and dried in an oven with air circulation (60 °C) until constant weight, for the determination of dry matter in kg of straw per hectare (Table 2). The LD in the pasture system (Table 2) was analyzed according to Rezende et al.^53^.

In order to estimate the daily amount of excreta, we considered the stocking rate adopted in the experiment (Table 2) and the values proposed by Haynes and Williams^54^. According to those authors, adult beef cattle can defecate on average 13 times a day and urinate 10 times a day, totaling a daily amount of 28.35 kg of feces and 19 L of urine.

The valuation was calculated based on the mean value of urea for the last 10 years in the fertilizer market^55–57^, namely $0.28 kg^−1^ ha^−1^ of urea, and considering the loss of nitrogen by volatilization, which according to Freney et al.^58^ and Subair et al.^59^ can reach up to 28%.

### Statistical analysis

The experiment was assembled in a randomized blocks design. The model adopted for the analysis of all response variables included the block’s and treatments fixed effects (3 blocks and 6 treatments), in addition to the random error. Statistical analysis were carried out by the function “dbc()” of the package “ExpDes.pt” of the software R Development Core Team^60^, and the mean values were compared by the Tukey’s test at a 5% probability level.

## Data Availability

Data are available from the corresponding author upon request.
